# Recombinant Protein Vaccines against Human Betacoronaviruses: Strategies, Approaches and Progress

**DOI:** 10.3390/ijms24021701

**Published:** 2023-01-15

**Authors:** Angelina Kovalenko, Ekaterina Ryabchevskaya, Ekaterina Evtushenko, Nikolai Nikitin, Olga Karpova

**Affiliations:** Department of Virology, Faculty of Biology, Lomonosov Moscow State University, 119234 Moscow, Russia

**Keywords:** human betacoronaviruses, SARS-CoV, MERS-CoV, SARS-CoV-2, COVID-19, recombinant protein vaccines, adjuvants, S protein, RBD, multi-epitope vaccines

## Abstract

Betacoronaviruses have already troubled humanity more than once. In 2002–2003 and 2012, the SARS-CoV and MERS-CoV, respectively, caused outbreaks of respiratory syndromes with a fatal outcome. The spread of the SARS-CoV-2 coronavirus has become a pandemic. These three coronaviruses belong to the genus Betacoronavirus and have a zoonotic origin. The emergence of new coronavirus infections in the future cannot be ruled out, and vaccination is the main way to prevent the spread of the infection. Previous experience in the development of vaccines against SARS and MERS has helped to develop a number of vaccines against SARS-CoV-2 in a fairly short time. Among them, there are quite a few recombinant protein vaccines, which seem to be very promising in terms of safety, minimization of side effects, storage and transportation conditions. The problem of developing a universal betacoronavirus vaccine is also still relevant. Here, we summarize the information on the designing of vaccines based on recombinant proteins against highly pathogenic human betacoronaviruses SARS-CoV, MERS-CoV and SARS-CoV-2.

## 1. Introduction

In December 2019, cases of pneumonia of unknown cause were reported in Wuhan, China [1]. It was soon discovered that the infectious agent was a new betacoronavirus, which was named as severe acute respiratory syndrome coronavirus 2 (SARS-CoV-2) by the International Committee on Taxonomy of Viruses [2]. In March 2020, the World Health Organization declared the outbreak of the coronavirus disease 2019 (COVID-19) a pandemic [3]. SARS-CoV-2 belongs to the family *Coronaviridae*, the representatives of which (SARS-CoV and MERS-CoV viruses) have previously caused outbreaks of high-mortality respiratory syndrome cases. In 2002–2003, there was an outbreak of severe acute respiratory syndrome (SARS) caused by the SARS-CoV virus in China. In 2012, cases of the Middle East respiratory syndrome (MERS) caused by the MERS-CoV virus were first reported in Saudi Arabia [4]. The MERS-CoV virus remains a concern, especially in Saudi Arabia and neighboring countries since individual cases of MERS are still being diagnosed [5]. A large number of vaccine candidates against SARS-CoV and MERS-CoV were developed. There are developments based on inactivated and live attenuated viruses, as well as protein vaccines, DNA-based vaccines, viral vector vaccines and virus-like particle (VLP) vaccines. Only a few vaccines against SARS and MERS have been subjected to clinical trials. These are an inactivated virus vaccine by Sinovac (Beijing, China) and a DNA vaccine by NIAID (Rockville, MD, USA) against SARS, and a DNA vaccine (GeneOne Life Science/Inovio Pharmaceuticals, Seoul, Republic of Korea/Plymouth Meeting, PA, USA) and several viral vector vaccines against MERS (MVA-MERS-S vaccine, CTC North GmbH & Co. KG, Hamburg, Germany; ChAdOx1 MERS vaccine, King Abdullah International Medical Research Center/University of Oxford, Riyadh, Saudi Arabia/Oxford, Great Britain; BVRS-GamVac-Combi vaccine, Gamaleya Research Institute of Epidemiology and Microbiology/Acellena Contract Drug Research and Development, Moscow/St. Petersburg, Russia) [6]. Currently, phase 1 of the clinical trials has begun for the inactivated SARS vaccine (no NCT ID) and for the DNA-based vaccine (NCT00099463) against SARS [6]. As far as vaccines against MERS are concerned, phase 1 (NCT02670187) and phase 1/2a (NCT03721718) of clinical trials have been completed for DNA-based MERS vaccine. Phase 1 of clinical trials was also completed for viral vector vaccines MVA-MERS-S (NCT03615911) and ChAdOx1 MERS (NCT04170829). The information about the 1/2 phase research (NCT04128059) for the BVRS-GamVac-Combi vaccine is on the site ClinicalTrials.gov (https://clinicaltrials.gov/ct2/show/NCT04128059, accessed on 5 January 2023). No vaccine against SARS and MERS has been approved for use.

To fight SARS-CoV-2, researchers focused their efforts on the development of various vaccines. Vaccine candidates include live-attenuated and inactivated vaccines, which have already become traditional, and also vaccines of new generations, such as recombinant protein vaccines, VLP vaccines, nucleic acid vaccines and vector vaccines [7]. In particular, mRNA vaccines, having not been previously applied in clinical practice, were approved. Two of them—Spikevax (Moderna, Cambridge, MA, USA) and Comirnaty (Pfizer/BioNTech, New York, NY, USA/Mainz, Germany)—became wildly used (https://covid19.trackvaccines.org/vaccines/approved/#vaccine-list, accessed on 5 January 2023). Both contain nucleoside-modified mRNA, which codes the S protein of SARS-CoV-2 and are delivered within lipid nanoparticles [6]. Vaccines that are based on a non-replicating adenovirus vector carrying the SARS-CoV-2 S protein coding sequence are also actively applied. A case in point is Sputnik V (Gamaleya, Moscow, Russia), Jcovden (Janssen (Johnson & Johnson), Leiden, Netherlands/New Brunswick, NJ, USA) and Vaxzevria (Oxford/AstraZeneca, Oxford, Great Britain/Cambridge, Great Britain/Södertälje, Sweden) (https://covid19.trackvaccines.org/vaccines/approved/#vaccine-list, accessed on 5 January 2023).

A large group of new generation COVID-19 vaccines are recombinant protein vaccines, which are the subject of this current review. These are vaccines that are considered to be among the most promising in terms of safety and side-effects avoidance. In addition to a high safety profile, recombinant protein vaccines have other advantages: they are more stable in comparison to mRNA vaccines and, in contrast to virus vector-based vaccines, potentially do not have application limitations concerning the use of the same vector in revaccination. Moreover, the production of protein recombinant vaccines does not require live virus cultivation since virus proteins, being vaccine components, are obtained in various recombinant expression systems. According to some research opinions, vaccines based on virus-like particles could also be included among the recombinant protein vaccines. However, in this review, the authors define “recombinant protein vaccines” as vaccines that contain isolated and purified coronavirus proteins and may otherwise be named “protein subunit vaccines”.

This review considers the principles of development and some examples of recombinant protein vaccines against human-pathogenic betacoronaviruses SARS-CoV, MERS-CoV and SARS-CoV-2. The review also presents the results of pre-clinical studies and clinical trials.

## 2. S Protein of Betacoronaviruses

Highly human-pathogenic coronaviruses SARS-CoV, MERS-CoV and SARS-CoV-2 belong to the genus *Betacoronavirus*, from the family *Coronaviridae*. SARS-CoV and SARS-CoV-2 belong to the subgenus *Sarbecovirus*, while MERS-CoV belongs to the subgenus *Merbecovirus*. Their genome (approximately 30 kb in size) is represented by positive-sense single-stranded RNA and encodes the following structural proteins: spike (S) protein, envelope (E) protein, membrane (M) protein and nucleocapsid (N) protein [8]. The S protein (spike protein) is a glycoprotein required for the virus to bind to receptors and enter the host cell. The M and E proteins are involved in the morphogenesis and assembly of virions, while the N protein plays a fundamental role in viral RNA packaging [9,10]. In addition to structural proteins, the betacoronavirus genome also encodes 16 non-structural proteins (Nsp1–16) and accessory ORFs proteins [8]. It is known that antibodies specific to the structural S and N proteins are detected in the serum of patients who have had COVID-19. A study by Jiang et al., (2020) also reported the detection of antibodies specific to the accessory ORF9b protein and non-structural Nsp5 protein [11]. The Nsp5 protein, also known as the 3CLpro protein, is the main protease of coronaviruses, a chymotrypsin-like enzyme [8,12]. The interaction of the accessory SARS-CoV-2 ORF9b protein with the human mitochondrial TOM70 protein inactivates type I interferon production [13]. However, of all the betacoronavirus proteins, the S protein appears to be the most promising antigen in the development of recombinant vaccines, since it induces the production of neutralizing antibodies that block the virus’ entry into the cell [10].

**Figure 1:**
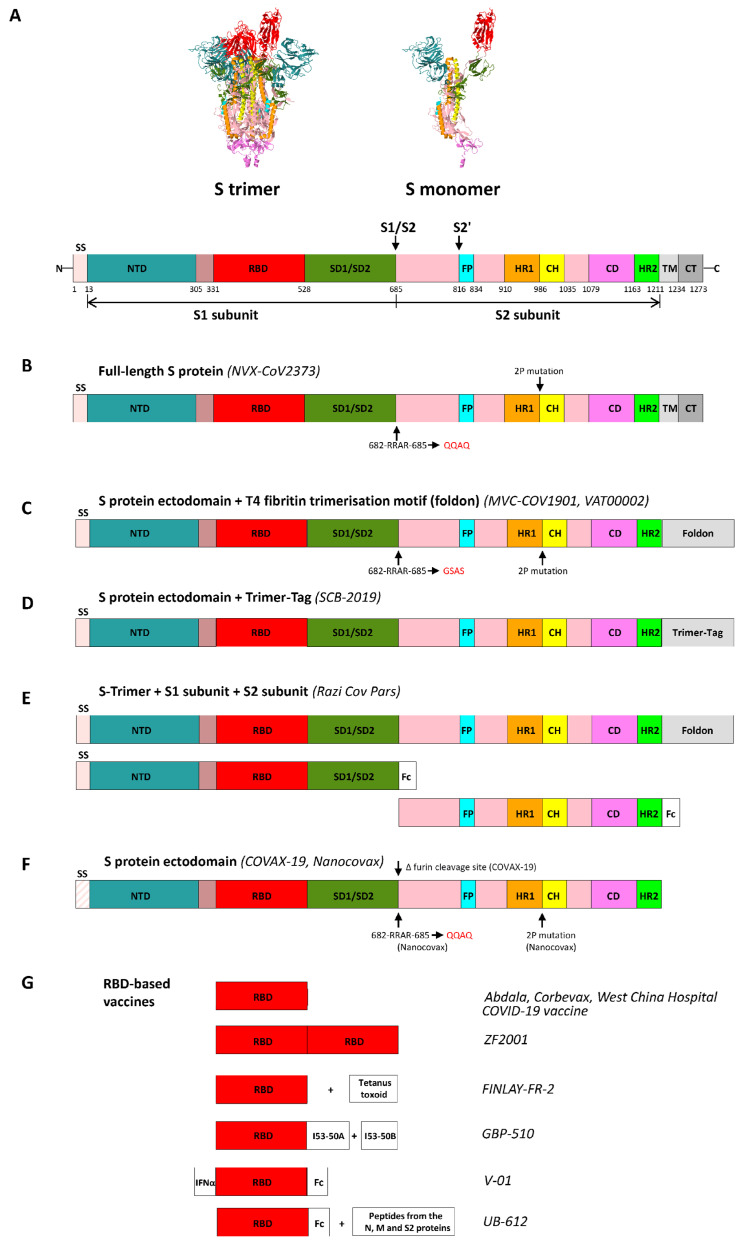
SARS-CoV-2 S protein structure (**A**) and graphical overview of design strategies of recombinant S protein-based COVID-19 vaccines, being at the phase 3 of clinical trials or approved (**B**–**G**). (**A**) SARS-CoV-2 S protein monomer and trimer (open state with one RBD in the “up” conformation) structural model (PDB code: 7DK3) [18,19] and linear diagram of the full-length S protein domain structure in the same color scheme. A structural model was obtained for the prefusion-stabilized trimerized S protein ectodomain using cryoelectronic microscopy. Protein structures were visualized using Jmol 14.31.34 software (http://www.jmol.org, accessed on 3 September 2022). (**B**) Antigen was based on full-length S protein within NVX-CoV2373 nanoparticle vaccine. (**C**,**D**) S protein ectodomain was supplemented with either T4 fibritin trimerization motif (foldon) (within MVC-COV1901 and VAT00002 vaccines) (**C**) or Trimer-Tag (within SCB-2019 vaccine) (**D**). (**E**) Antigens of Razi Cov Pars vaccine: S protein (trimerized by foldon) as well as S1 and S2 subunits, both fused with human IgG Fc at the C-terminus. (**F**) S protein ectodomain as an antigen of COVAX-19 vaccine (14–1213 amino acids are included) or Nanocovax vaccine (1–1213 amino acids are included). (**G**) The summary of antigens design within RBD-based vaccines: individual RBD monomer (Abdala, Corbevax, West China Hospital COVID-19 vaccine); RBD dimer (ZF2001); RBD monomer conjugated with tetanus toxoid (several RBD molecules are conjugated with on toxoid molecule) (FINLAY-FR-2); RBD fused with I53–50A protein component, which together with I53-50B component formed self-assembled nanoparticle, displaying 60 copies of RBD (GBP-510); RBD is armed with interferon-α (IFNα) at the N-terminus and is dimerized by human IgG1 Fc at the C-terminus (V-01); and RBD fused with single chain fragment crystallizable region (sFc) of a human IgG1 and supplemented with synthetic peptides of N-, M- and S2-proteins of SARS-CoV-2 (UB-612). SS: signal sequence; NTD: N-terminal domain; RBD: receptor-binding domain; SD1/SD2: subdomains 1 and 2; FP: fusion peptide; HR1: heptad repeat 1; CH: central helix; CD: connector domain; HR2: heptad repeat 2; TM: transmembrane domain; and CT: cytoplasmic tail. Two protease cleavage sites are indicated: the RRAR furin site (S1/S2) and the S2’ site. 2P mutation means the introduction of two proline mutations at residues 986 and 987.

The S protein contains 1255, 1353 and 1273 amino acids in SARS-CoV, MERS-CoV and SARS-CoV-2, respectively [14]. The SARS-CoV-2 S protein is 35% identical to the MERS-CoV S protein and 76% identical to the SARS-CoV S protein [15]. The S protein of betacoronaviruses contains an ectodomain consisting of two functional subunits, S1 and S2 (subunits 1 and 2), a transmembrane domain (TM) and a cytoplasmic tail (CT) [16]. The S1 subunit is required for the binding to a cellular receptor, which is represented by the angiotensin-converting enzyme 2 (ACE2) for SARS-CoV and SARS-CoV-2, and the dipeptidyl peptidase-4 (DPP4) molecule for MERS-CoV [4]. The S1 subunit (14–685 amino acid residues for SARS-CoV-2) contains an N-terminal domain (NTD), a receptor-binding domain (RBD) and subdomains 1 and 2 (SD1 and SD2). The S2 subunit (686–1211 amino acid residues for SARS-CoV-2) is required for the virus membrane fusion with the cell membrane; it contains the fusion peptide (FP), heptad repeat 1 (HR1), central helix (CH), connector domain (CD) and heptad repeat 2 (HR2) (Figure 1A) [17].

The S protein of betacoronaviruses is present in the virion as trimers (Figure 1A). It belongs to class I viral fusion proteins, along with the human immunodeficiency virus (HIV) envelope glycoprotein (Env) and influenza virus hemagglutinin (HA) [17]. For the virus to enter the cell, the S protein must be cleaved by cellular proteases into S1 and S2 subunits. Proteolytic activation of the SARS-CoV S protein occurs either after virion attachment to the cell surface due to the transmembrane serine protease 2 (TMPRSS2), or in the lysosome due to cathepsin proteases [20]. For SARS-CoV-2, this S protein activation occurs even at the stage of virion assembly in the Golgi apparatus of an infected cell, which is possibly due to the presence of a multibasic, furin-cleavage site (RRAR) at the S1/S2 junction. In a mature viral particle, the S1 and S2 subunits remain non-covalently associated [21,22]. This aspect distinguishes SARS-CoV-2 from SARS-CoV. MERS-CoV furin-cleavage site (RSVR) is similar to the one of SARS-CoV-2 [21]. Therefore, SARS-CoV-2 progeny virions with the pre-activated S protein are able to infect different cells in the body, which provides a high pathogenic potential for SARS-CoV-2 [23].

The betacoronaviruses S protein exists in two conformations: prefusion (‘before fusion’) and postfusion (‘after fusion’). The receptor-binding domain (RBD), which is part of the S1 subunit, is responsible for binding to the cellular receptor. In the context of the COVID-19 pandemic, structural studies of the SARS-CoV-2 S protein have become particularly relevant. The RBD mobility of the SARS-CoV-2 S protein in the prefusion conformation has been demonstrated using cryo-electron microscopy. Previously, seemingly stochastic RBD movements have been observed in the structural characterization of SARS-CoV and MERS-CoV [16]. It has been shown that, in the closed prefusion conformation, all three RBD trimers of the SARS-CoV-2 S protein are ‘down’ (lying down) and cannot bind to the ACE2 receptor. For the successful viral infection of the host cell, there should be at least one RBD ‘up’ (standing up) [23]. This S protein conformation is called open prefusion conformation. Some researchers believe that D614G mutation promotes the increase of the portion of the S protein in the open state with one RBD in the «up» conformation, which can explain the enhanced infectivity of the D614G variants of SARS-CoV-2 [22]. For SARS-CoV-2, it has been shown that its S protein also interacts with heparan sulfate, a negatively charged linear polysaccharide that is a component of many organs and tissues, via RBD. This interaction promotes the formation of the open S protein conformation required for binding to ACE2 [24]. According to a hypothetical scheme, the binding of one RBD up to ACE2 initiates the opening and binding of the RBD of the second, and then the third, S protein monomers to ACE2 molecules. As a result, S1 subunit shedding is induced and the S2’ sites within the S2 subunits that were previously deeply hidden in the spike are exposed [23,25]. Site-specific hydrolysis of the S protein occurs at the S2’ site immediately before the highly conserved S-F-I-E-D-L-L-F sequence [26]. In SARS-CoV-2, this process is catalyzed either by the TMPRSS2 protease in the case of virus penetration into cells through the cell membrane, or by lysosome cathepsins in the case of internalization by clathrin-mediated endocytosis. Cleavage at the S2’ site exposes the fusion peptide and dissociation of the S1 subunit that causes major conformational changes in the S2 subunit from prefusion to postfusion. The fusion peptide is propelled into the cell membrane, and the virus envelope and the cell membrane fuse [22]. Therefore, the interaction between an RBD and a specific cell surface receptor, which differs for different betacoronaviruses, causes major conformational changes in the S protein, which leads to the fusion of the coronavirus and host cell membranes.

## 3. Strategies for Obtaining Recombinant Protein Vaccines against Betacoronaviruses

There are three main strategies for the development of recombinant protein vaccines against a betacoronavirus infection. The first strategy is to use the full-length S protein as an antigen in the vaccine. It is believed that the prefusion conformation of the S protein trimer is the most optimal for inducing the maximum immune response, and it is proposed as one of the main targets for vaccine development. However, the prefusion state tends to be very unstable [27]. There are several protein engineering approaches that can solve this problem. The first approach is to introduce two proline residues (positions 986 and 987 for SARS-CoV-2) into the S2 subunit sequence between the heptad repeat 1 and the central helix. These mutations do not inhibit RBD motion in SARS-CoV-2 [27]. The other two approaches to stabilize the prefusion state of the isolated S protein are to remove the multibasic furin-cleavage site (SARS-CoV-2 and MERS-CoV) and replace the TM and CT domain sequences with an artificial motif of trimerization [28,29].

Earlier, in a study of the development of a SARS-CoV vaccine based on the full-length S protein, it was shown that an adverse effect, such as the antibody-dependent enhancement (ADE) of infection, was associated with the use of this vaccine [30]. The second strategy in the development of vaccines against betacoronavirus infections is to not use the full-length S protein as an antigen, but to instead use its fragments and exclude unwanted epitopes in the design of genetically engineered constructs. Thus, it has been proved for SARS-CoV, that antibodies specific to the S597-603 S1 subunit epitope are responsible for ADE [31]. In the classic case of ADE, specific IgGs form low affinity complexes with the virus, mediating its entering into cells of the immune system that displayed the FcγRII receptor [32]. The effect of ADE is associated with non-neutralizing antibodies or neutralizing antibodies at suboptimal concentrations [33]. Since the most robust virus-neutralizing antibodies are specific to RBD [34,35], scientists often use this antigenic determinant as a target when developing vaccines against betacoronaviruses. However, a recent study by Wan et al., (2020) described a new ADE mechanism, showing that a neutralizing monoclonal antibody (mAb) specific to MERS-CoV RBD mediated virus entry into immune cells *in vitro*. The mAb recognized the S protein RBD exclusively in the fusion-promoting ‘up’ orientation. Interaction with the S protein induced its conformational changes with the mAb binding to the Fc receptor, and the antibody/Fc receptor complex that functionally mimicked the viral receptor, mediated virus entry into immune cells. In addition, in the same study a neutralizing mAb specific for the SARS-CoV RBD was also shown to mediate the entry of the SARS-CoV pseudovirus. The authors of the study consider that the ADE of coronaviruses is mediated by neutralizing mAbs targeting the RBD of the S protein. They also demonstrated that for MERS-CoV entry into cells expressing both DPP4 and Fc receptors, there is a balance between DPP4-dependent and antibody-dependent entry pathways that can be determined by mAb dosages. A high dose of antibodies might help reduce the effect of ADE in MERS-CoV. In general, the researchers suggest that the ADE of viruses depends on the doses of antibodies, the level of expression of viral and Fc receptors in specific tissues, and some intrinsic characteristics of the antibodies [36]. Therefore, the dynamic nature of RBD should be taken into account when developing vaccines and therapeutics, and it can be assumed that the effect of ADE is less likely if antibodies target other regions of the S protein and do not cause changes in its conformation.

There is also an approach to engineering vaccines based on mapped linear B and T cell epitopes. For this purpose, partially overlapping short peptides are synthesized and immunogenic ones are identified among them. For example, Li et al., (2021) synthesized 211 such partially overlapping short peptide fragments and determined whether each peptide reacted with anti-SARS-CoV-2 antibodies in the blood sera of COVID-19 patients. This is how linear B epitopes of the S protein were identified. Two regions rich in linear epitopes were revealed: C-terminus of S1 subunit and a region close to the S2’ cleavage site and fusion peptide. The fact that there were almost no linear B epitopes in the RBD domain was an interesting result [37]. The mapping of T-epitopes is technically more difficult than B-epitopes. However, for SARS-CoV-2 proteins, the T-epitopes map has been made and very few CD4+ T cell epitopes have been shown in an RBD, while CD8+ T cell epitopes are evenly distributed among various coronavirus antigens [38]. When developing vaccines, a bioinformatic analysis is used to identify significant epitopes among all coronavirus proteins. Such multi-epitope vaccines are represented by peptides, which are corresponding the immunogenic epitopes of coronaviruses predicted by *in silico* methods, or their tandem repeats.

Recombinant antigens for protein vaccines can be produced in various expression systems, such as *Escherichia coli*, yeast, plants, insect cells and mammalian cells. *E. coli* is one of the earliest and most widely used expression systems for producing recombinant proteins. However, *E. coli* does not provide such post-translational modifications of expressed proteins such as glycosylation, which may affect the nature of the immune response and the formation of disulfide bonds, which affects the correctness of protein folding and, consequently, its solubility and stability [39]. It is nevertheless worth noting that He et al., (2021) characterized the structure of recombinant SARS-CoV-2 RBDs expressed in *E. coli* and mammalian HEK293 cells, and evaluated the ability of both recombinant proteins to bind to the ACE2 receptor, in their recent paper. The authors found no significant differences in the secondary and tertiary structure of proteins and their ability to bind to the receptor, despite the fact that the RBD expressed in *E. coli* was not glycosylated [40]. The surface of the S protein is known to be extensively shielded by glycans from antibody recognition, with the exception of the RBD, which explains the immunodominance of its epitopes [41]. At the same time, the protein yield in *E. coli* was 13.3 mg/L in bacterial culture and only 5 mg/L in mammalian cells [40]. *E. coli* as an expression system for recombinant betacoronavirus RBD has been successfully used in a number of studies [42,43]. Therefore, the protein produced in *E. coli*, which is a recombinant S protein RBD, can be used for the development of vaccines, along with proteins produced in other expression systems.

Insect cells are often used to express a recombinant antigen for a betacoronavirus vaccine. The Sf9 cell line obtained from the ovaries of the fall armyworm (*Spodoptera frugiperda*) [44] is very popular. Sf9 cells were used, for example, to express the recombinant antigen in the production of COVID-19 vaccines, such as the NVX-CoV2373 (full-length S protein vaccine) and the West China Hospital COVID-19 vaccine (RBD vaccine). However, for most current COVID-19 vaccines, recombinant proteins have been expressed in mammalian cell cultures, which, in recent years, have often been used for the production of various biopharmaceuticals, including antibodies and vaccine antigens. Cell lines such as HEK293 (human embryonic kidney 293) and its variants (293F and 293T), BHK cells (baby hamster kidney cells) and CHO cells (Chinese hamster ovary cells) are often used for the production of betacoronavirus vaccines. For example, recombinant antigens have been generated in CHO cells for the production of COVID-19 vaccines such as the MVC-COV1901 (S protein trimer vaccine) and ZF2001 (RBD dimer vaccine).

Since individual recombinant proteins usually elicit a weak immune response, most vaccines are developed in combination with various adjuvants that increase the immunogenicity of vaccines. There are adjuvants based on mineral salts, emulsions, microparticles, saponins, cytokines and chemokines, microbial components/products, liposomes, nucleic acids and nucleotides, polysaccharides, etc. However, only a few adjuvants are licensed for use in vaccines [45,46]. In pre-clinical studies of recombinant protein betacoronavirus vaccines, scientists have used various adjuvants, such as: Complete Freund’s Adjuvant (CFA); Incomplete Freund’s Adjuvant (IFA); Sigma Adjuvant System (SAS, an alternative to Freund’s adjuvant); monophosphoryl lipid A (MPL) in combination with trehalose dicorynomycolate (TDM); structurally modified plant viruses, etc. Table 1 shows examples of adjuvants in recombinant COVID-19 vaccines approved for clinical trials.

The selection of an adjuvant is an important stage in the development of effective and safe protein and inactivated vaccines. For example, a study by Honda-Okubo et al., (2015) showed that a delta inulin polysaccharide adjuvant not only increased the titer of neutralizing antibodies, but also reduced eosinophilic immunopathology in the lungs [54], an adverse vaccine-induced effect that was observed, for example, in the study of an inactivated SARS-CoV vaccine [55]. The data obtained in the study by Iwata-Yoshikawa et al., (2014) suggested that the same effect can be avoided when TLR agonist adjuvants are included in the vaccine [56].

## 4. Recombinant SARS and MERS Vaccines

### 4.1. Full-Length S Protein Vaccines

Full-length S protein recombinant vaccines have been developed against both SARS [30,57,58] and MERS [28].

In the study by He et al., (2006), the recombinant full-length S protein (FL-S) and its ectodomain (EC-S) (i.e., S protein without TM and CT domains) of the SARS-CoV virus were obtained. Recombinant proteins were expressed in insect cells and MPL and TDM was used as an adjuvant. The study demonstrated that both recombinant proteins induced high titers of neutralizing antibodies against SARS pseudoviruses constructed with the S proteins of Tor2, GD03T13 and SZ3, the representative strains of 2002 to 2003 and 2003 to 2004 human SARS-CoV and palm civet SARS-CoV, respectively [57].

It is known that when the recombinant ectodomain is expressed in eukaryotic systems, the resulting protein exists mainly in the monomeric form. Li et al., (2013) obtained a monomer of the SARS-CoV S protein ectodomain (S) and its trimerized form by combining the T4 fibritin trimerization motif (foldon) with the S protein ectodomain (S-foldon). To obtain secreted recombinant proteins, Sf9 insect cells were used in combination with a baculovirus expression system. In immunized mice, S-foldon induced a significantly higher titer of neutralizing antibodies than the monomeric S protein ectodomain. The authors also investigated the protective efficacy of recombinant proteins. Mice were immunized intramuscularly four times with S and S-foldon antigens in combination with an aluminum hydroxide gel and MPL adjuvant. After the fourth immunization, mice were intranasally infected with the SARS-CoV virus and were euthanized, and the level of viral load in the lungs was assessed. In groups of mice immunized with S and S-foldon antigens, the virus was not detected in the lungs, but was detected in the control group immunized with PBS buffer and adjuvant [58].

A similar approach using the T4 fibrin trimerization motif was used by Pallesen et al., (2017) to obtain a trimerized ectodomain of the MERS-CoV S protein (S-2P). The authors obtained a soluble trimerized S protein in the prefusion conformation that was stabilized due to two proline mutations (V1060P and L1061P) and a furin-cleavage site replacement (748-RSVR-751 -> ASVG). The recombinant protein expressed in mammalian 293F cells, in combination with the SAS adjuvant, following mice immunization induced a high titer of antibodies with neutralizing activity detected against various MERS-CoV pseudoviruses in an *in vitro* assay [28].

Kam et al., (2007) developed a recombinant vaccine (triSpike) based on a full-length S protein trimer expressed in BHK cells. The vaccine, in combination with an aluminum hydroxide gel adjuvant, induced the production of neutralizing antibodies against the SARS-CoV virus [30]. However, anti-S protein antibodies promoted the entry of the virus into B cells, thereby mediating the effect of ADE, which raised serious concerns about the use of the full-length S protein as a target for the SARS vaccine [30,59]. The authors of the study also note that it is especially necessary to unravel which viral epitopes or immunoglobulin isotypes are responsible for the enhanced infection. A possible strategy to prevent ADE is to use individual S protein fragments to obtain a vaccine antigen. Vaccines based on individual antigenic determinants of coronaviruses can be designed in such a way as to exclude the epitopes responsible for ADE (in particular, the aforementioned epitope S597-603 in the case of the SARS-CoV S protein).

### 4.2. RBD Vaccines

The attention of many investigators has been focused on the RBD, since it is this domain that mediates the interaction between the virus and the cellular receptor. Most neutralizing antibodies against coronaviruses target the S protein RBD and block its binding to the receptor [60]. There are many developments of recombinant protein SARS-CoV and MERS-CoV vaccines based on the S protein RBD. These vaccines induce the production of high titers of neutralizing antibodies, while no pronounced immunopathological effects associated with the use of such vaccines were reported [42,61,62,63].

Du et al., (2009) expressed recombinant SARS-CoV RBDs (rRBDs) in various expression systems, such as mammalian (293T), insect (Sf9) and *E. coli* cells, comparing the rRBDs obtained when testing their efficacy in a mouse model. All three rRBD proteins retained their native conformation. When administered with the SAS adjuvant, they induced a potent immune response and protected mice against SARS-CoV, neutralizing antibodies suppressed virus replication in lung tissues. The authors concluded that any of the expression systems presented in the study could be used to produce recombinant protein rRBD vaccines against SARS [42].

In the study performed by Lan et al., (2014) the immunogenicity of a recombinant protein vaccine based on the MERS-CoV rRBD obtained using a baculovirus expression system in combination with various adjuvants in mice was tested. Intramuscular injection of the rRBD in combination with aluminum hydroxide and CpG ODN mediated the induction of RBD-specific humoral and cellular immunity. The rRBD in combination with IFA and CpG ODN, after subcutaneous injection, induced potent RBD-specific antibodies and T cell response, but the level of neutralizing antibodies was low [64]. A vaccine candidate based on the MERS-CoV rRBD, in combination with aluminum hydroxide, induced a high level of neutralizing antibodies and T cell immunity in rhesus monkeys, and also alleviated the course of pneumonia and reduced the viral load during MERS-CoV infection [65].

The trimerized RBD-Fd protein obtained through MERS-CoV RBD fusion with the T4 fibritin trimerization motif was described in the paper by Tai et al., (2016). Mammalian 293T cells were used as the expression system. The RBD-Fd bound to DPP4, a molecule acting as a receptor for MERS-CoV, induced the production of strong RBD-specific neutralizing antibodies in mice, and in combination with an aluminum-based adjuvant, protected transgenic mice (with human dipeptidyl peptidase 4 (hDPP4)) from death when infected with MERS-CoV [63].

A paper by Dai et al., (2020) described a strategy for obtaining the dimeric form of the MERS-CoV RBD and the dimeric form of the SARS-CoV RBD, coined tandem repeat single chain dimer (sc-dimer) by the authors. Mammalian HEK293T cells were used as the expression system. Mice were immunized with the recombinant protein in combination with the AddaVax adjuvant (similar to MF-59). It was shown that the dimeric form of RBD, compared to the monomeric form, had greater immunogenicity, and induced significantly higher titers of neutralizing antibodies [66].

One approach to the development of vaccines is the production of fusion proteins containing the target antigen and the fragment crystallizable region (Fc) of human immunoglobulin G (IgG). The presence of the Fc domain increases the plasma half-life of the protein, which prolongs its therapeutic activity [67] and leads to slower renal clearance for large molecules [68]. The Fc domain promotes the proper folding of the fusion protein and can improve the solubility and stability of the partner molecule [69]. The attached Fc domain also enables the interaction of fusion proteins with the Fc receptors (FcRs) located on immune cells, which is particularly important for their use in vaccines [70]. An approach using the human IgG1 Fc fragment was taken to develop a number of RBD-based SARS and MERS vaccines. RBD-Fc was shown to induce neutralizing antibodies against SARS-CoV in immunized rabbits and mice [61]. Du et al., (2007) showed that, after immunization with RBD-Fc, neutralizing antibodies could persist for 12 months, protecting most immunized mice against SARS-CoV [62]. A similar approach using the Fc fragment was also applied in the development of MERS-CoV vaccines [71,72,73,74,75,76]. In particular, Zhang et al., (2016) conducted a study to determine the adjuvant that would most optimally increase the immunogenicity of the S377-588-Fc protein (the MERS-CoV RBD fused to the Fc fragment). Thus, the authors compared several commercially available adjuvants, including Freund’s adjuvant, aluminum hydroxide, MPL, Montanide ISA51 and MF59, in terms of their ability to increase the immunogenicity of the developed recombinant protein vaccine. MF59 was shown to be the most potent since it induced the highest titers of IgG1 and IgG2a antibodies and neutralizing antibodies. A significant enhancement of the S377-588-Fc antigen immunogenicity after the MF59 adjuvant addition and the protection of immunized mice with hDPP4 against MERS-CoV after infection led the authors to suggest that MF59 was the optimal adjuvant for MERS-CoV RBD-based vaccines [75].

### 4.3. S Protein Fragment Vaccines

Recombinant protein vaccines based on the S1 subunit demonstrated immunogenicity and a protective effect against betacoronaviruses. Li et al., (2013), who have been mentioned during the discussion of vaccines based on the full-length S protein, also obtained recombinant S1 and S1-foldon proteins, which contributed to the production of neutralizing antibodies and along with full-length S and S-foldon proteins, provided protection against SARS-CoV in mice [58]. Wang et al., (2015) demonstrated that a recombinant protein based on the MERS-CoV S1 subunit, in combination with an aluminum phosphate adjuvant, induced a high titer of neutralizing antibodies and protected non-human primates against severe lung disease after MERS-CoV infection [77]. A study by Adney et al., (2019) reported on a protein vaccine based on the MERS-CoV S1 subunit used in combination with Advax HCXL and SAS adjuvants. The recombinant vaccine provided alpacas with complete protection against MERS-CoV that correlated with high serum titers of neutralizing antibodies. Lower titers of neutralizing antibodies were found in dromedary camels, but virus replication in immunized animals was limited mainly to the nasal turbinate; this contrasts with non-immunized animals, in which MERS-CoV spread deeper through the respiratory tract into the trachea [78].

Kim et al., (2020) developed several variants of the MERS vaccine based on the S1 subunit fused to the foldon trimerization motif. The sequences of either the RS09 peptide (mimics lipopolysaccharide and is TLR4 agonist) or flagellin (TLR5 agonist) were added to the constructs. Recombinant proteins were expressed in the HEK293 cells. The immunogenicity of vaccine candidates was tested in mice using two methods of vaccine administration: traditional subcutaneous injection and using a dissolving microneedle array, which is a plate with carboxymethylcellulose-based needles loaded with a recombinant antigen. The study authors reported that the administration of vaccines using microneedles elicited a more effective humoral immune response and more efficient production of neutralizing antibodies than a subcutaneous injection, regardless of the presence of additional TLR binding sites in the antigen sequence. However, the inclusion of the RS09 sequence led to an enhanced immune response, and a significant induction of neutralizing antibodies, when compared with the antigen without RS09 when administered subcutaneously. Interestingly, the inclusion of the flagellin sequence did not affect the immunogenicity of subcutaneously administered vaccines [79].

Jiaming et al., (2017) obtained a recombinant N-terminal domain (rNTD), which is part of the S1 subunit of the MERS-CoV S protein. The MERS-CoV rNTD, in combination with aluminum hydroxide and CpG, was shown to induce potent cellular immunity and antigen-specific neutralizing antibodies in mice. Moreover, the rNTD demonstrated protection against MERS-CoV infection in transgenic mice with hDPP4 that was comparable to the rRBD, resulting in a reduced pulmonary pathology. Thus, rNTD is another potential antigen for MERS vaccines [80].

Some studies have focused on the development of S2 subunit vaccines, but no positive results have been obtained regarding the ability of these vaccines to elicit neutralizing antibodies or induce protective reactions [58]. Guo et al., (2005) showed that the recombinant fragment of the SARS-CoV S2 subunit (amino acid residues 681–980) expressed in *E. coli* cells was specifically recognized by the blood serum of people who had had SARS. In combination with the IFA adjuvant, the recombinant protein induced a Th2-dominant response in mice, but no neutralizing antibody titer was detected [81]. However, some monoclonal antibodies to the highly conserved HR1 and HR2 domains of the S2 subunit are known to have a broad spectrum of neutralizing activity against various SARS-CoV isolates [82,83]. This indicates the potential of the S2 subunit as a target for the development of a universal vaccine against various betacoronaviruses.

### 4.4. Vaccines Based on Non-S Structural Proteins

Although most papers focus on vaccines based on the S protein or its fragments, there are also data relating to the development of vaccines based on the structural N and M proteins. The SARS-CoV N protein expressed in *Nicotiana benthamiana* induced IgG1 and IgG2a antibodies in mice, with CFA used for the first immunization and IFA used for the second immunization. No adjuvants were used during the third and fourth doses of the vaccine. The induction of a cellular immune response was also shown [84]. In the study by Liu et al., (2006), the recombinant SARS-CoV nucleocapsid protein (rN) expressed in E. coli, in combination with Montanide ISA51 and CpG adjuvants, induced mainly IgG2a isotype antibodies in immunized mice. In contrast, after immunization with the rN protein in PBS without adjuvants, the antibodies produced were mainly of the IgG1 isotype. These results showed that immunization with the rN protein in combination with Montanide ISA51 and CpG adjuvants elicited a Th1-biased immune response [85]. In a study by He et al., (2005), two synthetic peptides corresponding to the main immunodominant epitopes of the SARS-CoV M protein (M1-31 and M132-161) were produced. Both peptides induced high antibody titers in immunized rabbits [86]. Thus, the immunogenicity of SARS and MERS protein vaccines based on N and M proteins has been demonstrated, but the protective efficacy of these vaccine candidates is unknown. It is possible to use N and M proteins in the development of vaccines, but for full protection against coronavirus infection, it is necessary to obtain virus-neutralizing antibodies that are produced only when immunized with the S protein or its individual fragments.

### 4.5. Multi-Epitope Vaccines

The use of bioinformatic approaches for SARS and MERS suggested the design of multivalent vaccines containing T and B cell epitopes of S, E, M, N and ORFs proteins. Using *in silico* methods, the selected epitopes were tested for the possibility of their molecular interaction with alleles of the major histocompatibility complex (HLA, human leucocyte antigens). A stable molecular interaction with the TLR2 and TLR4 receptors for SARS-CoV protein epitopes and with the TLR3 receptor for MERS-CoV protein epitopes was also predicted. Probably, the decision of the study authors to analyze for molecular interaction with certain TLRs could be explained by the presence of different adjuvant sequences: a truncated *Onchocerca volvulus* activation-associated secreted protein-1 and human β-defensin within SARS and MERS vaccines, respectively. Nevertheless, it is likely that in both cases, more than one or two types of the TLR as well as others pattern-recognition receptors could contribute to activation of innate immune mechanisms. The analysis of the nucleotide sequence encoding the predicted epitopes showed that this sequence was able to provide a high level of expression in mammalian cells. The authors believe that, since vaccines contain T- and B-cell epitopes, they should induce both cellular and humoral immune responses [87,88]. However, the results of *in vivo* trials of these vaccines are currently not publicly available. Bioinformatic methods enable vaccine developers to analyze the coronavirus proteome and based on the information obtained, select the most immunogenic peptides, which can help in the design of a potentially effective vaccine candidate in a fairly short time, the immunogenicity and protectivity of which should be tested in animal models.

## 5. Recombinant COVID-19 Vaccines

Amid the pandemic of the novel coronavirus infection, there is a need to create effective COVID-19 vaccines. Due to the COVID-19 pandemic, the number of SARS-CoV-2 vaccines that have been developed and that have been subjected to clinical trials, is far greater than the number of SARS and MERS vaccines. The results of the development of recombinant SARS and MERS vaccines have become the starting point for the creation of a relevant vaccine against the novel coronavirus infection. As of October 25, 2022, 18 of the 49 COVID-19 vaccines approved for use are recombinant protein vaccines (https://covid19.trackvaccines.org/). Most of the vaccines being developed and vaccines at different stages of pre-clinical and clinical studies include the S protein or its RBD domain as an antigen. Figure 1B–G illustrates the approaches used to develop COVID-19 vaccines (approved for phase 3 clinical trials or already approved for use) based on the SARS-CoV-2 S protein and its fragments. Table 2 shows examples of some recombinant protein COVID-19 vaccines.

### 5.1. Full-Length S Protein Vaccines

Novavax (USA) has developed a recombinant NVX-CoV2373 (Nuvaxovid) vaccine based on prefusion-stabilized S protein trimers assembled into nanoparticles. Protein stability was achieved by replacing the furin-cleavage site (682-RRAR-685 -> QQAQ) and introducing two proline mutations (K986P and V987P) (Figure 1B). The recombinant protein was expressed in Sf9 insect cells. The Matrix-M adjuvant was used in the vaccine [89]. Successful trials in animal models made it possible to conduct clinical trials of the vaccine, which confirmed the efficacy and safety of NVX-CoV2373 [90]. The NVX-CoV2373 vaccine provides 89.7% (according to the published results of phase 3 clinical trials conducted in the UK: EUCTR2020-004123-16 and NCT04583995) [91] and 92.6% (according to the results of phase 3 clinical trials conducted in the USA and Mexico: NCT04611802) [92] protection against the SARS-CoV-2 infection. The NVX-CoV2373 vaccine has been approved for use in 40 countries (October 25, 2022).

The MVC-COV1901 vaccine, developed by the Medigen Vaccine Biologics Corporation (Taiwan) with the support of the NIAID (USA), is represented by a prefusion-stabilized trimerized ectodomain (S-2P protein) (amino acid residues 1–1208). In order to improve antigen stability, two substitutions of K986P and V987P were made, and the furin-cleavage site was mutated (682-RRAR-685 -> GSAS) at the S1/S2 junction (Figure 1C). The T4 fibrin trimerization motif, HRV3C protease cleavage site, octa-histidine tag and TwinStrep96 tag (tags necessary for the affinity purification of the recombinant protein, which were then removed using the HRV3C protease), were added to the sequence. The protein was expressed in mammalian CHO cells. The recombinant protein is used in the vaccine in combination with the CpG 1018 (Dynavax, USA) and aluminum hydroxide adjuvant [93]. Phase 2 clinical trials (NCT04695652) have shown that the MVC-COV1901 vaccine induces high titers of neutralizing antibodies, is safe, is well-tolerated and rarely causes a fever in both young and elderly people [94]. Phase 3 clinical trials are ongoing (NCT05426343, NCT05198596 and NCT05011526); the MVC-COV1901 vaccine has already been approved for use in four countries (Eswatini, Paraguay, Somaliland and Taiwan).

The COVAX-19 (SpikoGen) vaccine (Vaxine Pty Ltd./CinnaGen Co., Australia/Iran) is based on the S protein ectodomain (ECD) with the removed furin-cleavage site (Figure 1F) expressed in Tni insect cells. It is interesting that according to the authors, the study of the engineered recombinant antigen by molecular dynamics simulation showed its ability to form a stable trimer, despite the absence of transmembrane and cytoplasmic domains and a trimerization motif. The Advax-CpG55.2 adjuvant is used in the vaccine. Pre-clinical studies have demonstrated that the vaccine induced high titers of neutralizing antibodies and the formation of CD4+ and CD8+ memory T cells in mice [95]. Immunized hamsters infected with SARS-CoV-2 showed a decreased viral load in the oropharynx, the nasal turbinate and lungs [96]. Data from phase 1 (NCT04453852) and phase 2 (NCT04944368) clinical trials have shown that the COVAX-19 vaccine has an acceptable safety and tolerability profile, without serious side effects [97]. The COVAX-19 vaccine is currently undergoing phase 3 clinical trials (NCT05005559, NCT05542862 and NCT05175625) and is approved for use in Iran.

The Razi Cov Pars vaccine (Razi Vaccine and Serum Research Institute, Iran), which is at the stage of phase 3 clinical trials (IRCT20201214049709N3), has also been approved for use in Iran. This vaccine differs in that it simultaneously contains three immunogenic components: S1 and S2 subunits fused to the Fc fragment of human IgG (expressed in Expi293F human cells) and the trimerized SARS-CoV-2 S protein (expressed in ExpiCHO-S™ Cells) (Figure 1E). The RAS-01 adjuvant (Razi Adjuvant System-01) is an oil-in-water emulsion comprised of sesame, olive and soybean oils, as well as the non-ionic surfactant Tween 80 (Polysorbate 80). The vaccine’s effectiveness was evaluated in various animal models. The vaccine induced the production of neutralizing antibodies. The protective effect has also been shown in experiments with the SARS-CoV-2 infection. The vaccination schedule is worthy of note. There were two intramuscular injections and one intranasal injection of the vaccine [98].

Trimer-Tag technology (C-propeptide of human type I(α) collagen) makes it possible to express recombinant trimerized proteins in mammalian cells and purify them using affinity chromatography. The S trimer, in combination with AS03 (GlaxoSmithKline, United Kingdom) or CpG 1018 (Dynavax, USA) and aluminum hydroxide as adjuvants, induced high levels of neutralizing antibodies and a Th1-mediated cellular immune response in model animals, and also protected non-human primates against the SARS-CoV-2 infection [99]. Phase 1 clinical trials (NCT04405908) found that both variants of the SCB-2019 vaccine (with added AS03 or CpG and aluminum hydroxide) elicited robust humoral and cellular immune responses against SARS-CoV-2, with high virus-neutralizing activity [100]. In phase 2/3 clinical trials (NCT04672395), the SCB-2019 vaccine was used in combination with the CpG 1018 and aluminum hydroxide adjuvant. Two doses of the SCB-2019 vaccine were shown to provide protection against SARS-CoV-2, with an average efficacy of 67.2% [101]. The SCB-2019 vaccine is currently undergoing phase 3 clinical trials (NCT05188677 and NCT05470803).

The Nanocovax vaccine, by Nanogen Pharmaceutical Biotechnology JSC (Vietnam), is represented by the recombinant SARS-CoV-2 S protein ectodomain in the prefusion conformation; the stabilization of which was achieved by K986P and V987P substitutions and mutations in the furin-cleavage site (682-RRAR-685 -> QQAQ) (Figure 1F). The protein was expressed in CHO cells, and aluminum hydroxide acts as an adjuvant in the vaccine. The vaccine’s safety and efficacy were demonstrated on three model organisms (BALB/c mice, Syrian hamsters and *Macaca leonina* primates). The vaccine induced high levels of S protein-specific IgG and neutralizing antibodies. When studying the protective efficacy of the vaccine in hamsters, it was also shown that it protected the upper respiratory tract against the SARS-CoV-2 infection [102]. According to the data from phase 1/2 clinical trials (NCT04683484), no serious side effects were reported in the study participants after vaccination. The Nanocovax vaccine induced the production of S protein-specific and virus-neutralizing antibodies [103]. The Nanocovax vaccine is currently undergoing phase 3 clinical trials (NCT04922788).

Sanofi Pasteur (France) in collaboration with GlaxoSmithKline (United Kingdom), developed a SARS-CoV-2 recombinant protein vaccine VAT00002 (VAT00008, Vidprevtyn) based on a prefusion-stabilized S protein trimer (K986P and V987P substitutions were made, 682-RRAR-685 was replaced by GSAS, and the T4 fibrin trimerization motif was added) (Figure 1C). A prefusion-stabilized S protein trimer was produced in the baculovirus expression system and the vaccine formulation includes an AS03 adjuvant [104]. The preliminary results of phase 1/2 clinical trials (NCT04537208) were unexpected, in that the vaccine failed to elicit an effective immune response in people over 60 years of age and a higher-than-expected reactogenicity was noted after the second vaccination. A study of the final drug substance characteristics revealed that the level of residual proteins of the producer cells was higher than had been stated before the start of clinical trials, meaning that the antigen concentration in the vaccine formulation was significantly lower (about four to six times) than planned. By the time this was discovered, the study participants had received at least one dose of the vaccine. Thus, in order to continue clinical trials of the vaccine, it was necessary to optimize its composition [105]. Further data from phase 2 clinical trials showed that the vaccine had an acceptable immunogenicity, safety and reactogenicity profile, including in people aged 60 years and older and those highly susceptible to diseases (NCT04762680) [106]. The vaccine being developed by Sanofi/GSK is currently undergoing phase 3 clinical trials. The NCT04904549 study is registered on the *ClinicalTrials.gov* website and has two stages. At the first stage, a monovalent vaccine is evaluated, which contains the S antigen of the SARS-CoV-2 D614G variant; at the second stage, a bivalent vaccine is evaluated, which includes the S protein of the D614G variant and the S protein of the SARS-CoV-2 beta variant (B.1.351).

The PIKA COVID-19 Vaccine, developed by Yisheng Biopharma (China), contains a prefusion-stabilized trimerized S protein ectodomain expressed in CHO cells, and the PIKA acts as an adjuvant. The T4 fibrin trimerization motif was added to the nucleotide sequence encoding the target protein; K986P and V987P substitutions were made to stabilize the protein in the prefusion conformation; 682-RRAR-685 was replaced by GSAS in the furin-cleavage site. Pre-clinical studies have shown that the vaccine induces sustained cellular and humoral immune responses, as well as the production of neutralizing antibodies, which remain at a high level for at least 400 days. A study of the protective efficacy of the vaccine in non-human primates showed that vaccine-induced neutralizing antibodies that protected against virus replication in the lungs of animals after the SARS-CoV-2 infection [107]. The vaccine is undergoing phase 2/3 clinical trials (NCT05463419).

Another example of a trimerized S protein ectodomain vaccine is the ZR-202-CoV (202-CoV) vaccine, developed by Shanghai Zerun Biotechnology/Walvax Biotechnology (China), which is undergoing phase 2 clinical trials (NCT04990544). The ectodomain was trimerized by including the T4 fibrin trimerization motif in the sequence. K986P and V987P substitutions, as well as the replacement of 682-RRAR-685 with GGSG at the furin-cleavage site, contributed toward protein stabilization in the prefusion conformation. The recombinant protein was produced in CHO cells, and it is used in the vaccine in combination with an adjuvant based on aluminum hydroxide and CpG 7909. Pre-clinical studies have shown that the vaccine induced the formation of CD4+ T cells and the production of neutralizing antibodies in both mice and primates, and neutralizing antibodies remained at a high level for at least six months. Studies on the protective efficacy of the vaccine have demonstrated a reduction in the viral load and inflammation in the lungs of immunized Syrian golden hamsters after the SARS-CoV-2 infection [108].

The SpFN COVID-19 vaccine, developed by the US Army Medical Research and Development Command (USA), which is undergoing phase 1 clinical trials (NCT04784767), is represented by *Helicobacter pylori* ferritin nanoparticles containing the S protein ectodomain region (amino acid residues 12–1158), modified by the introduction of two proline residues (K986P and V987P) and the furin-cleavage site replacement (682-RRAR-685 -> GSAS). The combination of the antigen with ferritin ensures the formation of trimers. Stabilization of trimers formation on the ferritin molecule was achieved by introducing mutations (P1143S, F1148I, Y1155I, F1156H) into the sequence of the C-terminal heptad repeat of the S protein, to increase the coiled coil interactions. Expi293F human cells were selected as the expression system; the vaccine contains the ALFQ adjuvant [109]. Pre-clinical studies have demonstrated the immunogenicity of the vaccine, its ability to induce the production of neutralizing antibodies and the formation of T cells [110,111]. A study in primates showed the protective effect of the vaccine, specifically the rapid elimination of the virus from the respiratory tract and lungs after infection with high doses of SARS-CoV-2 [111].

Ambiguous results were obtained in phase 1 clinical trials of the Sclamp vaccine (University of Queensland, Australia) based on a trimerized S protein ectodomain stabilized using a ‘molecular clamp’. The ‘molecular clamp’ is a trimerization motif comprising fragments of the HIV-1 gp41 glycoprotein (amino acid residues 540–576 and 619–656), which form a stable six-helix bundle. The inclusion of these sequences makes it possible to obtain a trimerized S protein stabilized in the prefusion conformation [150]. In general, this recombinant vaccine, in combination with the MF59 adjuvant, elicited a strong immune response, but the gp41 glycoprotein sequences present in the ‘clamp’ created obstacles for the diagnostic test for HIV, which prevented the further use of this vaccine [151], so the project was discontinued.

### 5.2. RBD Vaccines

Given that the effect of ADE was observed when using SARS vaccines based on the full-length S protein [30,59], scientists tend to use individual fragments of the S protein when developing COVID-19 vaccines in order to minimize possible adverse immunopathology. The S protein RBD is an attractive target in the development of such vaccines. The Abdala vaccine (CIGB-66) (Center for Genetic Engineering and Biotechnology, Cuba) is approved for use in six countries and is an example of a vaccine based on the RBD monomer (Figure 1G) expressed in *P. pastoris* yeast cells. The vaccine contains an aluminum hydroxide-based adjuvant. The results of phase 1/2 clinical trials (RPCEC00000346) have shown that the vaccine is safe, well-tolerated and induces a humoral immune response [112]. The Abdala vaccine is undergoing phase 3 clinical trials (RPCEC00000359).

Since the RBD immunogenicity is rather limited, Dai et al., (2020), using the already mentioned sc-dimer strategy that was applied to develop the MERS-CoV RBD dimer and the SARS-CoV RBD dimer, with the support of Anhui Zhifei Longcom Biopharmaceutical (China), developed the ZF2001 (Zifivax) vaccine against COVID-19, which is a SARS-CoV-2 RBD dimer (Figure 1G). Pre-clinical studies have shown that the dimeric form of the SARS-CoV-2 RBD induces a 10- to 100-fold increase in neutralizing antibody titers compared with the monomer [66]. The recombinant vaccine protein is expressed in CHO cells and used in combination with an aluminum hydroxide adjuvant. Phase 1 (NCT04445194) and phase 2 (NCT04466085) clinical trials have shown that the vaccine does not cause serious side effects. After three-stage vaccination, the level of seroconversion of neutralizing antibodies reached 93–100%. The vaccine also induced a cellular immune response, as demonstrated by the balanced production of cytokines associated with Th1- and Th2-cells [113]. The results of phase 3 clinical trials (NCT04646590) have shown that the vaccine is safe and effective against COVID-19 for at least six months after full vaccination [114]. Phase 3 clinical trials are ongoing (NCT05128643, NCT05091411 and NCT05107375). The ZF2001 vaccine against COVID-19 is approved for use in China, Colombia, Indonesia and Uzbekistan.

The FINLAY-FR-2 (Soberana 02) vaccine, developed by the Instituto Finlay de Vacunas (Cuba), is approved for use in four countries (Cuba, Iran, Nicaragua and Venezuela). This is a vaccine based on multiple copies of recombinant RBD (expressed in CHO cells) that is conjugated with tetanus toxoid to maintain its stability (Figure 1G). Aluminum hydroxide is used as an adjuvant. The vaccine has demonstrated immunogenicity in pre-clinical studies [115]. According to the published results of phase 1 and phase 2a clinical trials, the Soberana 02 vaccine is safe and elicits an immune response in people aged 19–80 years and it induces neutralizing antibodies and a specific T-cell response [116]. The results of phase 3 clinical trials have shown the effectiveness of two doses of the Soberana 02 vaccine to be at 71.0%, while the addition of the Soberana Plus vaccine as a third dose (SARS-CoV-2 RBD dimer in combination with aluminum hydroxide) increases the effectiveness to 92.4% (IFV/COR/09) [117].

The Corbevax (BECOV2A) vaccine, which is manufactured by the Indian company Biological E. Limited and is approved for use in Botswana and India with an aluminum hydroxide and CpG 1018 adjuvant, can be mentioned as an example of the successful expression of recombinant RBD (Figure 1G) in *P. pastoris* yeast cells. Mutations that reduce glycosylation (deletion of the N331 residue) and aggregation (C538A substitution) were introduced into the RBD sequence. The recombinant RBD219-N1C1 protein obtained was equivalent to the wild-type recombinant RBD protein (RBD219-WT) in an *in vitro* ACE-2 binding assay. The vaccine was shown to induce high levels of antigen-specific IgG and neutralizing antibodies in mice. When using the vaccine in combination with aluminum hydroxide, a Th2-biased immune response was noted. Therefore, when testing the vaccine in clinical trials, the CpG 1018 adjuvant was added to its composition, in addition to aluminum hydroxide, to induce a preferable Th1 immune response [118,119]. Data from phase 1/2 clinical trials (CTRI/2020/11/029032) have confirmed the immunogenicity and safety of the vaccine [120]. Phase 3 clinical trials (CTRI/2021/08/036074) evaluated the effectiveness of the Corbevax vaccine in comparison with the Covishield adenovirus vector vaccine (ChAdOx1 nCoV-19, Oxford–AstraZeneca COVID19 vaccine) (United Kingdom), which is licensed in many countries. According to the study results, both vaccines have a comparable safety profile and anti-RBD IgG seroconversion rate. In addition, Corbevax induced higher titers of neutralizing antibodies [121].

The GBP510 (SKYCovione) vaccine (SK Bioscience Co., Ltd., Republic of Korea, jointly with the University of Washington (USA)) was developed based on self-assembling protein nanoparticles that expose 60 copies of the RBD in order to increase its immunogenicity (Figure 1G). The protein nanoparticle model (I53-50) was developed using computational design. It is an icosahedral nanoparticle constructed from trimers (I53-50A) and pentamers (I53-50B). The RBD domain was linked to the I53-50A component by a glycine–serine linker, and this construct was expressed in human Expi293F cells. To initiate the assembly of nanoparticles, the recombinant RBD-I53-50A protein obtained was mixed with an I53-50B pentamer isolated from *E. coli*. The vaccine contains the AS03 adjuvant. Pre-clinical studies have shown that the vaccine induces an effective humoral response. It was found that nanoparticle-specific antibodies were also produced, but their level was approximately comparable with, or slightly lower than, the level of RBD-specific antibodies produced. Vaccine-induced neutralizing antibody titers were ten times higher than those induced by the prefusion-stabilized trimerized S protein ectodomain [122]. The vaccine’s protective efficacy was confirmed in rhesus monkeys. In animals given two doses of the vaccine, anti-nanoparticle antibody titers positively correlated with anti-S antibody titers and viral neutralizing activity, suggesting that pre-existing anti-nanoparticle antibody responses do not affect antigen-specific antibody responses during boosting [123]. The vaccine was approved for clinical trials, which demonstrated its immunogenicity and good tolerability [124]. The GBP510 vaccine is currently undergoing phase 3 clinical trials (NCT05007951 and NCT05501522) and is approved for use in the Republic of Korea.

The West China Hospital COVID-19 vaccine (West China Hospital, Sichuan University, China), presented by an RBD (Figure 1G), expressed in Sf9 insect cells and having an aluminum hydroxide adjuvant, has been shown to be effective in pre-clinical studies, inducing a humoral response in immunized mice, rabbits and non-human primates. Furthermore, the sera of immunized animals had a virus-neutralizing impact on both the SARS-CoV-2 pseudovirus and live SARS-CoV-2 virus in *in vitro* assays. The vaccine has also demonstrated its protective efficacy in experiments on primates with the SARS-CoV-2 infection [128]. Successful pre-clinical studies have enabled clinical trials of the vaccine and it is currently undergoing phase 3 clinical trials (NCT04904471 and NCT04887207).

A study by Marlin et al., (2021) reported on the αCD40.RBD vaccine candidate, which is an anti-human CD40 humanized 12E12 IgG4 antibody C-terminally fused to the SARS-CoV-2 RBD. This construct makes it possible to target the antigen at dendritic cells via the CD40 surface receptor. The immunogenicity of the αCD40.RBD vaccine co-administered with the Poly-IC adjuvant (polyinosinic-polycytidylic acid) was shown in humanized mice in which the vaccine elicited specific T and B cell responses. It was also shown that a single dose of the vaccine administered without an adjuvant was sufficient to significantly increase the titers of neutralizing antibodies in macaques that had been infected with SARS-CoV-2 six months earlier. Vaccine-induced antibodies were shown *in vitro* to cross-neutralize various SARS-CoV-2 variants, including D614G, B1.1.7 and, to a lesser extent, B1.351 [144].

As in the case of SARS and MERS, developers use approaches to develop COVID-19 vaccines based on the combination of an RBD and Fc fragment. The V-01 vaccine (Livzon Mabpharm Inc, China) is a hybrid vaccine in which the RBD is fused to IFNα at the N-terminus and to the Fc fragment of human IgG1 at the C-terminus, due to which dimerization occurs (Figure 1G). The construct also includes the PADRE epitope (pan HLA DR-binding epitope) to enhance the response of CD4+ T cells [152]. The recombinant protein is expressed in mammalian CHO cells and used with aluminum hydroxide as an adjuvant [125]. Pre-clinical studies have shown the protective efficacy of the vaccine candidate, which has made it possible to proceed to clinical trials of the vaccine [126]. The results of phase 1 clinical trials have shown the vaccine’s immunogenicity, its ability to induce high titers of neutralizing antibodies and its safety and good tolerability [125]. The V-01 vaccine is currently undergoing phase 3 clinical trials and has been shown to induce sustained humoral immunity in study participants vaccinated with the inactivated vaccine (NCT05096832) [127]. It has been approved for use in China.

The RBD and Fc fusion approach was also applied in the AKS-452 vaccine (University Medical Center Groningen, the Netherlands), which is undergoing phase 2/3 clinical trials (CTRI/2021/10/037269). The protein is expressed in CHO cells and the vaccine contains the Montanide ISA 720 adjuvant. The vaccine’s protectivity was confirmed in pre-clinical studies [129]. Data from phase 1/2 clinical trials (NCT04681092) have also shown its immunogenicity and a favorable safety profile [130].

Kentucky Bioprocessing (USA) used a unique approach in the production of the KBP-201 vaccine, consisting of the chemical conjugation of the recombinant RBD-Fc antigen expressed in *N. benthamiana* with tobacco mosaic virus (TMV) virions modified by the introduction of reactive lysine into the N-terminal region of the coat protein. Before conjugation, the modified viral particles were exposed to ultraviolet radiation to inactivate the viral RNA. Pre-clinical studies of the vaccine’s immunogenicity have demonstrated that it induces an effective immune response in immunized mice, both with and without the CpG 7909 adjuvant. The vaccine without an adjuvant induced a balanced Th1/Th2 response, while the vaccine with an adjuvant induced a Th1-biased response. Both vaccine variants induced the production of virus-neutralizing antibodies [131]. The protective efficacy of the vaccine was proven in studies with humanized mice expressing the human angiotensin-converting enzyme 2 (hACE2). The vaccine protected the mice from death after the SARS-CoV-2 infection [132]. The KBP-201 vaccine (with the CpG adjuvant) is undergoing phase 1/2 clinical trials (NCT04473690).

In a study by Siriwattananon et al., (2021), the RBD-Fc fusion protein was obtained by fusing the SARS-CoV-2 RBD with the Fc domain of human IgG1. The genetic construct encoding the fusion protein was cloned into a geminivirus vector for expression in *N. benthamiana* plants. Plant-produced RBD-Fc, together with aluminum hydroxide as an adjuvant, elicited an effective humoral and cellular response in mice and macaques (*Macaca fascicularis*) [134]. The vaccine protectivity was demonstrated in transgenic mice with hACE2, monkeys and Wistar rats [135]. The study was supported by Baiya Phytopharm Co Ltd. (Thailand); the Baiya SARS-CoV-2 Vax 1 vaccine is currently undergoing phase 1 clinical trials (NCT04953078).

The development of the recombinant Betuvax-CoV-2 vaccine (Human Stem Cell Institute, Russia), based on the RBD and SD1 domain (important for RBD mobility) of the SARS-CoV-2 virus fused to the Fc fragment of human IgG1, was described by Krasilnikov et al., (2022). The antigen is expressed in CHO cells, adsorbed on a plant-derived adjuvant, betulin (pentacyclic triterpenoid) that forms spherical particles (betuspheres). The authors showed that the vaccine induced the production of high titers of antibodies (RBD-specific and SARS-CoV-2-neutralizing) and the development of T cell immunity in mice [133]. The Betuvax-CoV-2 vaccine is undergoing phase 1/2 clinical trials (NCT05270954).

Ma et al., (2020) developed *H. pylori* ferritin nanoparticles that conjugated with the RBD and conserved fragments of the S2 subunit, heptad repeats (HR) (RBD-HR nanoparticles) and RBD-conjugated ferritin nanoparticles (RBD nanoparticles), using SpyTag/SpyCatcher technology. SpyTag (ST, peptide comprising13 amino acids) reacts with the SpyCatcher protein (SC, protein comprising138 amino acids) to form an intermolecular isopeptide bond. In order to obtain nanoparticles for the vaccine, SC ferritin was expressed in *E. coli*, while ST-RBD and ST-HR were expressed in CHO-S cells. The incubation of SC ferritin with ST antigens leads to irreversible conjugation of ferritin and antigens and the formation of nanoparticles. In combination with the SAS adjuvant (an alternative to Freund’s adjuvant), RBD-HR nanoparticles were found to induce humoral and cellular responses after two immunizations of mice. Both types of nanoparticles protected transgenic mice with hACE2 from the SARS-CoV-2 infection, but only RBD-HR nanoparticles induced the formation of neutralizing antibodies not only to SARS-CoV-2, but also to other coronaviruses. The induction of the production of antibodies to ferritin was also shown [146]. The study suggests that *H. pylori* ferritin and its antibodies are likely not to be toxic *in vivo*, since two influenza vaccines based on *H. pylori* ferritin nanoparticles are known to have been clinically evaluated (NCT03186781 and NCT03814720) and no serious side effects have been reported. Nevertheless, this issue needs to be investigated and discussed further.

A vaccine candidate based on three recombinant proteins (Co1, PE and CoF) was designed and analyzed by Kovalenko et al. (2022) [43]. The Co1 protein contains a sequence corresponding to the SARS-CoV-2 RBD (amino acid residues 319–541, GenBank accession number: YP_009724390.1). The polyepitope (PE) protein contains highly conserved fragments of the SARS-CoV-2 S2 subunit (amino acid residues 950–1041 and amino acid residues 1157–1210) that include previously predicted partially overlapping T and B cell epitopes [15,153]. The CoF protein contains the RBD and the S2 subunit epitope (amino acid residues 1182–1210). Recombinant proteins were expressed in *E. coli* cells. Structurally modified spherical particles (SPs) formed during tobacco mosaic virus (TMV) thermal remodeling are used in the vaccine as an adjuvant. The authors suggested that SPs function as an antigen depot. The vaccine candidate immunogenicity was demonstrated in mice. The titers of antibodies to coronavirus antigens were significantly higher when immunized with compositions than when immunized with individual antigens. In this case, the immune response was produced mainly to coronavirus antigens and not to the adjuvant. Pre-clinical studies showed serum neutralizing activity against SARS-CoV-2 in hamsters immunized with the vaccine candidate [43].

### 5.3. S Protein Fragment Vaccines

Kim et al., (2020) developed recombinant protein vaccines against SARS-CoV-2 based on the S1 subunit and the S1 subunit fused to the RS09 sequence, using the approach that was used for MERS vaccine engineering, with a microneedle array being employed for transdermal vaccine administration. The vaccines developed induced the effective production of antigen-specific antibodies in mice as soon as two weeks after immunization, but the inclusion of the RS09 adjuvant did not significantly affect the effectiveness of vaccination [79].

Ren et al., (2020) evaluated the immunogenicity of a recombinant protein based on the SARS-CoV-2 S1 subunit fused to the Fc fragment (S1-Fc) in preclinical studies. The protein was expressed in CHO cells. Rabbits were immunized with the S1-Fc protein and the AD20Gold+ adjuvant (nanoemulsion with synthetic MPL). For non-human primates, CFA was used for the first immunization and AD11.10 (saponin-based microemulsion) was used for the second immunization. The recombinant S1-Fc protein was shown to induce high antibody titers. The sera of immunized rabbits and primates had a high level of virus-neutralizing activity [143].

It is believed that the S2 subunit is less immunogenic than the S1 subunit since it is more strongly shielded by N-glycans and less accessible for interaction with the immune system. The S2 subunit cannot induce an effective humoral response; however, due to the high conservation of its sequence among different coronaviruses, it is a target for cross-reactive antibodies and CD4+ T cells that recognize SARS-CoV-2 and other coronaviruses [41]. Therefore, due to its high conservation, the S2 subunit may be of interest in terms of developing a universal coronavirus vaccine.

### 5.4. Vaccines Based on Non-S Structural Proteins

The N, M and E protein sequences are similar for SARS-CoV, MERS-CoV and SARS-CoV-2, which may indicate their potential as vaccine targets for a universal betacoronavirus vaccine. Several T cell epitopes have been identified in these proteins in previous studies on SARS-CoV and MERS-CoV [154], so their inclusion in a SARS-CoV-2 vaccine may help enhance the T cell response and improve cross-protection [41]. The development of the Convacell vaccine based on the N protein (St. Petersburg Research Institute of Vaccines and Sera, Russia), which is expressed in *E. coli* [141], has also been reported. The recombinant protein obtained has immunogenic properties. It is used with an adjuvant based on squalane, α-tocopherol and polysorbate 80 [142]. The protective properties of the recombinant N protein were studied in Syrian hamsters. It has been reported that, in immunized animals after the SARS-CoV-2 infection, a decrease in the severity of lung damage was noted. The vaccine is undergoing phase 1/2 clinical trials (NCT05156723). In mass studies, the approach to the N protein vaccine development was not widely accepted. However, the N protein can be used as an additional vaccine antigen. The N protein is highly conserved among coronaviruses in comparison to the S protein, and although N protein vaccines generally do not induce neutralizing antibodies, its use in a universal coronavirus vaccine may contribute to the induction of T cell immunity.

### 5.5. Multi-Epitope Vaccines

A different approach to the development of COVID-19 vaccines should be considered separately. This relates to multi-epitope vaccines. The approach is based on a bioinformatic analysis, which makes it possible to identify the antigenic determinants of SARS-CoV-2 T and B cell epitopes. T cell epitopes that elicit a long-term immune response of CD4+ and CD8+ T cells are most often analyzed. The prediction of B cell epitopes (both linear and conformational) is considered to be less reliable, and they do not induce a strong humoral response [155]. Potential T cell epitopes can be predicted for both structural and non-structural proteins; however, in most cases, the S protein is analyzed as the main target of coronavirus vaccines. The use of *in silico* methods optimizes the vaccine development process, allowing only immunogenic T and B cell epitopes of the coronavirus to be included in its composition for effective stimulation of cellular and humoral responses. Multi-epitope vaccines have the potential to help avoid the adverse effect of ADE. Epitope selection should be made using rational filtering criteria based on the SARS-CoV-2 biology, which include predicted immunogenicity, location of epitopes, glycosylation sites and polymorphic sites. Candidate epitopes that are located within or adjacent to functional domains with evidence of antibody-mediated viral neutralization should be selected [156]. Some examples of such vaccines will be discussed below.

Some investigators have presented designs of multi-epitope vaccines developed using bioinformatic methods, but these vaccines’ *in vitro* and *in vivo* efficacy is currently unknown [157,158,159]. Using bioinformatic methods, Enayatkhani et al., (2020) engineered a multi-epitope vaccine based on T and B cell epitope-enriched N, ORF3a and M (NOM) protein domains that could potentially induce a CD4+ and CD8+ T cell immune response. A molecular dynamics simulation confirmed the stability of the engineered NOM protein complexes with the TLR4 innate immunity receptor and the HLA-A*11:01 cellular immunity receptor [157]. Kalita et al., (2020) presented a vaccine design consisting of 18 CTL (cytotoxic T lymphocyte), 6 HTL (helper T lymphocyte) and 9 B cell epitopes of SARS-CoV-2 N, M and S proteins connected by linkers and a human β-defensin sequence (an adjuvant). The molecular dynamics simulation confirmed the vaccine construct stability [158]. Sarkar et al., (2020) used computational experiments to analyze the sequences of N, M, ORF3a and S proteins of the SARS-CoV-2 virus and selected T and B cell epitopes of the N and S proteins for inclusion in the vaccine construct, which also contains the PADRE sequence, the universal epitope AKFVAAWTLKAAA. The results of molecular docking conducted to determine whether all selected epitopes can bind to HLA class I and II molecules were positive [159].

Coleon et al., (2022) demonstrated the immunogenicity and efficacy of a bioinformatically engineered vaccine in pre-clinical studies. The authors used *in silico* methods to identify conserved T and B cell epitopes of the S and N proteins, highly homologous among 38 sarbecoviruses, to develop a recombinant protein vaccine (CD40.CoV2) targeting antigens to dendritic cells through the CD40 surface receptor. For this purpose, they used an anti-human CD40 humanized 12E12 IgG4 antibody. Mammalian CHO-S cells were selected for the vaccine construct expression. Poly-IC was used as an adjuvant since this adjuvant enhances APC maturation. Immunization with CD40.CoV2 elicited high levels of antibodies with cross-neutralizing activity against SARS-CoV-2 (Wuhan, *α, β, γ, δ* and *κ*) and SARS-CoV variants in transgenic mice with hACE2, which was associated with survival after the SARS-CoV-2 infection. The ability of CD40.CoV2 to induce the response of cytotoxic memory T cells was also demonstrated [145].

Multi-epitope vaccines have also shown their efficacy in clinical trials. The EpiVacCorona vaccine by the Vector State Research Center of Virology and Biotechnology (Russia) consists of short peptides carrying conserved linear B cell epitopes of the SARS-CoV-2 S protein, the choice of which was based on the published spatial structures of the SARS-CoV S protein and data on the SARS-CoV-2 genetic sequences. The following SARS-CoV-2 S protein epitopes are used in the vaccine (overlapping parts are in bold):

1. ^454^RLFRKSNLKPFERDISTEIYQAGS^477^—epitope from the RBD region;
2. ^1181^KEIDRLNEVA**KNLNESLIDLQE**^1202^—epitope from the heptad repeat 2 (HR2) region;
3. ^1191^**KNLNESLIDLQE**LGKYEQYIK^1211^—epitope from the HR2 region.

Based on these epitopes, peptides for the vaccine were chemically synthesized, then conjugated with a carrier protein (a fusion product of the maltose-binding protein and the SARS-CoV-2 N protein) and adsorbed on aluminum hydroxide [136]. Pre-clinical studies have shown that the vaccine accelerates virus elimination from the upper respiratory tract in ferrets and prevents the development of pneumonia after SARS-CoV-2 infection in hamsters and lower primates [137]. The EpiVacCorona vaccine has passed phase 1/2 (NCT04527575) [138] and phase 3 (NCT05021016 and NCT04780035) clinical trials. It has been approved for use in Russia, Cambodia, Turkmenistan and Venezuela.

The UB-612 peptide vaccine (COVAXX and United Biomedical Inc. Asia, USA/Taiwan) contains the RBD fused to the Fc domain of human IgG1, produced in CHO cells and synthetic immunogenic peptides based on highly conserved sequences of the S2 subunit of the S protein, N and M proteins (Figure 1G). The vaccine also includes a peptide derived from the measles fusion protein, to enhance the immune response (patented UBITh1a^®^ technology) and a CpG and aluminum phosphate adjuvant (AdjuPhos^®^) [139]. In phase 1 (NCT04545749 and NCT04967742) and phase 2 (NCT04773067) clinical trials, the vaccine demonstrated a favorable safety profile, efficacy against Delta and Omicron variants and prolonged B and T cell immunity [140]. The UB-612 vaccine is undergoing phase 3 clinical trials (NCT05293665).

The CoVac-1 vaccine, developed by the University of Tübingen (Germany), which is undergoing phase 1/2 clinical trials (NCT04954469), is a vaccine of chemically synthesized six peptides based on T cell epitopes of the N, S, E, M and ORF8 proteins. Synthetic lipopeptide XS15 emulsified in Montanide ISA51 VG was used as an adjuvant. The results of phase 1 trials (NCT04546841) showed no serious side effects after vaccination. In all study participants, the vaccine induced a T cell response mediated by the Th-1 and cytotoxic T cells [53].

Here, we looked at 25 COVID-19 recombinant protein vaccines at different stages of clinical trials. Primarily, these vaccines are based on SARS-CoV-2 S protein (10 vaccines) or its receptor-binding domain (RBD) (11 vaccines). To solve the well-known problem of RBDs low immunogenicity, various approaches such as the dimerization of RBD, its fusion to the human IgG Fc-fragment or the designing of nanoparticles displaying multiple RBD copies were used. The vaccines effectiveness was demonstrated in clinical trials. For both S protein-based and RBD-based vaccines, there are examples of the high efficacy achievement: a 92.6% and 92.4% efficacy was revealed for NVX-CoV2373 (S protein-based) and Soberana 02 Plus (RBD-based), respectively. Therefore, both the S protein and RBD seem to be promising antigens for vaccine development. The main argument in favor of the S protein use is keeping the recombinant antigen state corresponding to the native one. The researchers’ interest in an RBD-based vaccine is perhaps driven by the ease of the RBD production procedure compared to the full-size S protein and is probably due to the concerns about undesirable immune reactions previously observed during the study of SARS-CoV vaccines based on the full-size S protein. Nevertheless, clinical trials data evidenced the appropriate safety for all types of COVID-19 vaccines; the observed side effects were minor. Furthermore, three examples of multi-epitope vaccines and the example of the vaccine based on the SARS-CoV-2 N protein were considered in the current review. At present, these approaches to the development of coronavirus vaccines are not widespread, though they could be helpful for the further design of a universal vaccine against coronaviruses.

The above-cited results of the various recombinant protein vaccine developments against COVID-19 studies demonstrate the relevance and highly promising potential of this technological platform.

## 6. Conclusions

The development of COVID-19 vaccines remains a crucial task since vaccination is the most effective way to prevent infectious diseases and protect the population from epidemics. Since the beginning of the COVID-19 pandemic, the development of recombinant protein vaccines has received less attention than, for example, the development of nucleic acid vaccines or vector vaccines. However, recombinant protein vaccines have certain advantages, primarily a high safety profile. Recombinant protein vaccines can also serve as a useful supplement to two-stage (prime-boost) vaccination. Finally, recombinant protein vaccines, unlike mRNA and viral vector vaccines, are less demanding of the production, storage and transportation processes.

Most COVID-19 vaccines are based on the inclusion of either the full-length S protein or its individual fragments, in particular the RBD domain, in their composition as a vaccine antigen. Previous experience in the development of protein vaccines against SARS has shown that the use of vaccines based on the full-length S protein may be associated with an undesirable effect, such as ADE. Therefore, when creating COVID-19 vaccines, developers try to use individual fragments of the S protein while the focus is shifted to the RBD, the main target of neutralizing antibodies. Approaches using structural N, M and E proteins as vaccine antigens have not been widely used; however, due to the conservation of these proteins, they can be used as additional antigenic determinants in order to induce a T cell response by the vaccine. Furthermore, using *in silico* approaches, multi-epitope vaccines containing the main T and B cell epitopes of coronaviruses have been developed, while unwanted epitopes that may potentially cause various immunopathologies can be excluded when engineering a vaccine antigen. The inclusion of highly conserved regions of various human pathogenic coronaviruses in a multi-epitope vaccine may be an important step toward the development of a universal vaccine, since the risk of new coronavirus infections in the future remains. Therefore, the presence of a panel of vaccines obtained using various approaches can provide effective protection against COVID-19, and the use of structural protein regions that are conserved for the three main betacoronaviruses (SARS-CoV, MERS-CoV and SARS-CoV-2) may help develop a universal vaccine that will protect not only against SARS, MERS and COVID-19, but also against other betacoronaviruses that are not yet circulating in the human population.

Antigens for recombinant protein vaccines against COVID-19 have been produced in different expression systems, with more traditional systems such as mammalian or insect cells being particularly popular. However, some developers use alternative approaches, such as the use of tobacco plants as an expression system of recombinant antigens for a vaccine.

The use of suitable adjuvants in recombinant protein vaccines makes it possible to solve the problem of the low immunogenicity of these vaccines. As can be seen from the examples presented in this review, protein vaccines against COVID-19 include a variety of adjuvants, and all these vaccines have proved to be quite successful. Since different adjuvants have different mechanisms of action, it is rather difficult to compare protein vaccines in terms of efficacy. The correct choice of an adjuvant is important for creating an effective, but also a safe vaccine, since it is important to prevent the development of a Th2-biased immune response, which is associated with the development of pulmonary immunopathologies [160]. Even vaccines that have already been approved for clinical trials may show ambiguous results, and projects may be suspended or even terminated. However, only the development and testing of a wide range of vaccines, including recombinant protein vaccines, will make it possible to control the situation with the incidence of COVID-19.

## Figures and Tables

**Table 1:** Some adjuvants used in recombinant protein COVID-19 vaccines approved for clinical trials *.

Adjuvant	Composition	Main Mechanism of Action
Aluminum compounds	Aluminum hydroxide, aluminum phosphate, etc.	Depot effect, enhancement of phagocytosis of the antigen and activation of the pro-inflammatory NLRP3 pathway [39]
CpG oligodeoxynucleotides (CpG ODN)	Synthetic oligodeoxynucleotides containing unmethylated CpG repeats	Toll-like receptor 9 (TLR9) agonists [45]
Monophosphoryl lipid A (MPL)	Detoxified lipopolysaccharide derivative of gram-negative bacterium *Salmonella minnesota*	TLR4 agonist [47]
Montanide ISA51 and 720	Emulsions consisting of oils and monooleate mannide surfactant	Antigen depot, recruitment of antigen-presenting cells (APCs) and induction of lymphocyte migration into draining lymph nodes [48]
MF59	Squalene-based emulsion adjuvant	Induction of cytokines and chemokines, recruitment of immune cells [45]
Advax	Delta inulin adjuvant	Activation of the complement alternative pathway, enhancement of humoral and cellular immune responses with the lack of induction of pro-inflammatory cytokines production [49]
Matrix-M	Saponin adjuvant	Induction of leukocyte migration into draining lymph nodes [50]
AS03	Oil emulsion containing squalene, α-tocopherol and polysorbate 80	Action similar to the MF59 adjuvant, as well as increased antigen uptake, in particular by monocytes, antigen presentation in draining lymph nodes [46]
PIKA	Synthetic chemical analogue of dsRNA, polyinosinic-polycytidylic acid with kanamycin and calcium	TLR3 agonist [51]
ALFQ	Liposomes containing saponin QS-21 and synthetic monophosphoryl lipid A (3D-PHAD^®^)	MPL is a TLR4 agonist, saponin QS21 activates NLRP3 [52]
XS15	Synthetic lipopeptide	TLR1 and TLR2 agonist [53]

* There are currently no recombinant protein SARS and MERS vaccines approved for clinical trials.

**Table 2:** Several recombinant protein vaccines against COVID-19 in clinical trials and pre-clinical studies as of 25 October 2022.

Developer	Vaccine Name	Antigen/Technology	Target Antigen Expression System	Type of Adjuvant	Clinical Phase	Approvals	References
Full-length S protein-based vaccines							
Novavax (Gaithersburg, MD, USA)	NVX-CoV2373 (Nuvaxovid*)*	Nanoparticle vaccine composed of trimeric full-length S proteins	Sf9 insect cells	Matrix-M	3 (EUCTR2020-004123-16, NCT04583995, NCT04611802, NCT05249816, NCT05556720, NCT05463068 and NCT05372588)	40 countries (USA, European countries and others)	[89,90,91,92]
Medigen Vaccine Biologics Corporation (Taipei, Taiwan)/NIAID (Rockville, MD, USA)/ Dynavax (Emeryville, CA, USA)	MVC-COV1901	Prefusion-stabilized trimeric S protein (S-2P)	CHO cells	CpG 1018 (Dynavax) and Al(OH)3	3 (NCT05426343, NCT05198596 and NCT05011526)	Eswatini, Paraguay, Somaliland and Taiwan	[93,94]
Vaxine Pty Ltd. (Adelaide, Australia)/ CinnaGen Co. (Tehran, Iran)	COVAX-19 (SpikoGen)	S protein ectodomain	Tni insect cells	Advax-CpG55.2 (Advax-SM)	3 (NCT05005559, NCT05542862 and NCT05175625)	Iran	[95,96,97]
Razi Vaccine and Serum Research Institute (Karaj, Iran)	Razi Cov Pars	S-Trimer, S1 and S2 subunits	Expi293F human cells, ExpiCHO-S™ Cells	RAS-01 (Razi Adjuvant System-01)	3 (IRCT20201214049709N3)	Iran	[98]
Clover Biopharmaceuticals Inc. (Shanghai, China)/ GSK (Brentford, United Kingdom)/Dynavax (Emeryville, CA, USA)	SCB-2019	S-Trimer (Trimer-Tag technology)	CHO cells	AS03(GSK) or CpG 1018 and Al(OH)3	3 (NCT05188677 and NCT05470803)	-	[99,100,101]
Nanogen Pharmaceutical Biotechnology JSC (Ho Chi Minh City, Vietnam)	Nanocovax	Prefusion-stabilized S protein	CHO cells	Aluminium hydroxide	3 (NCT04922788)	-	[102,103]
Sanofi Pasteur (Lyon, France)/ GSK (Brentford, United Kingdom)	VAT00002 (VAT00008, Vidprevtyn)	Prefusion-stabilized trimeric S protein	expresSF^+^ insect cells	AS03 (GSK)	3 (NCT04904549)	-	[104,105,106]
Yisheng Biopharma (Beijing, China)	PIKA COVID-19 Vaccine	Prefusion-stabilized trimeric S protein	CHO cells	PIKA	2/3 (NCT05463419)	-	[107]
Shanghai Zerun Biotechnology (Shanghai, China) / Walvax Biotechnology (Kunming, China)	ZR-202-CoV (202-CoV)	Prefusion-stabilized trimeric S protein	CHO cells	CpG 7909 and Al(OH)3	2 (NCT04990544)	-	[108]
US Army Medical Research and Development Command ( Frederick, MD, USA)	SpFN COVID-19 Vaccine	Spike-ferritin nanoparticle	Expi293F cells	ALFQ	1 (NCT04784767)	-	[109,110,111]
**RBD-based vaccines**							
Center for Genetic Engineering and Biotechnology (CIGB) (Havana, Cuba)	Abdala (CIGB-66)	Monomeric RBD	*P. pastoris* yeast	Aluminium hydroxide	3 (RPCEC00000359)	Cuba, Mexico, Nicaragua, Saint Vincent and the Grenadines, Venezuela and Vietnam	[112]
Anhui Zhifei Longcom Biopharmaceutical (Hefei, China)/ Institute of Microbiology, Chinese Academy of Sciences (Beijing, China)	ZF2001 (Zifivax)	Tandem-repeat dimeric RBD	CHO cells	Aluminium hydroxide	3 (NCT04646590, NCT05128643, NCT05091411 and NCT05107375)	China, Colombia, Indonesia and Uzbekistan	[66,113,114]
Instituto Finlay de Vacunas (Havana, Cuba)	FINLAY-FR-2 (Soberana 02)	Conjugated vaccine (RBD and tetanus toxoid)	CHO cells	Aluminium hydroxide	3 (IFV/COR/09)	Cuba, Iran, Nicaragua and Venezuela	[115,116,117]
Biological E. Limited (Hyderabad, India)	Corbevax (BECOV2A)	Recombinant RBD	*P. pastoris* yeast	CpG 1018 (Dynavax) and Al(OH)3	3 (CTRI/2021/08/036074)	Botswana and India	[118,119,120,121]
SK Bioscience Co., Ltd. (Seongnam, Republic of Korea)	GBP510 (SKYCovione)	Self-assembled two-component nanoparticle vaccine displaying RBD	Expi293F cells	AS03 (GSK)	3 (NCT05007951 and NCT05501522)	Republic of Korea	[122,123,124]
Livzon Mabpharm Inc (Zhuhai, China)	V-01	IFN-PADRE- RBD-Fc dimer	CHO cells	Aluminium hydroxide	3 (NCT05096832)	China	[125,126,127]
West China Hospital, Sichuan University (Chengdu, China)	West China Hospital COVID-19 vaccine	Recombinant RBD	Sf9 insect cells	Aluminium hydroxide	3 (NCT04904471 and NCT04887207)	-	[128]
University Medical Center Groningen (Groningen, Netherlands)	AKS-452	RBD-Fc	CHO cells	Montanide^TM^ ISA 720	2/3 (CTRI/2021/10/037269)	-	[129,130]
Kentucky Bioprocessing (Owensboro, KY, USA)	KBP-201	RBD-Fc	*N.benthamiana* plants	CpG	1/2 (NCT04473690)	-	[131,132]
Human Stem Cell Institute (Moscow, Russia)	Betuvax-CoV-2	RBD-SD1-Fc	CHO cells	Betulin	1/2 (NCT05270954)	-	[133]
Baiya Phytopharm Co Ltd. (Bangkok, Thailand)	Baiya SARS-CoV-2 Vax 1	RBD-Fc	*N.benthamiana* plants	Aluminium hydroxide	1 (NCT04953078)	-	[134,135]
**Multi-epitope vaccines**							
Vector State Research Center of Virology and Biotechnology (Koltsovo, Russia)	EpiVacCorona	Chemically synthesized peptide immunogens of the SARS-CoV-2 S protein conjugated to a carrier protein (SARS-CoV-2 N protein and bacterial maltose-binding protein)	*E. coli* for carrier protein expression	Aluminium hydroxide	3 (NCT05021016 and NCT04780035)	Russia, Cambodia, Turkmenistan and Venezuela	[136,137,138]
COVAXX (Hauppauge, NY, USA)/United Biomedical Inc. Asia (Taipei, Taiwan)	UB-612	RBD-Fc and peptides representing conserved epitopes from the N-, M- and S2 proteins	CHO cells for RBD-Fc expression	UBITh1a^®^, CpG and Aluminum phosphate	3 (NCT05293665)	-	[139,140]
University Hospital Tübingen (Tübingen, Germany)	CoVac-1	Synthetic peptides from the S-, N-, M-, E- and ORF8 proteins	-	XS15 emulsified in Montanide ISA51 VG	1/2 (NCT04954469)	-	[53]
**Vaccines Based on Non-S Structural Proteins**							
St. Petersburg Research Institute of Vaccines and Sera (Saint Petersburg, Russia)	Convacell	N protein	*E. coli*	Squalane, α-tocopherol and polysorbate 80	1/2 (NCT05156723)	-	[141,142]
**Several vaccines in pre-clinical studies**							
Developer	Antigen/technology		Target antigen expression system	Type of adjuvant		Developments based on the same approach	References
Univ. of Pittsburgh (Pittsburgh, PA, USA)	Microneedle array delivered S1 subunit		HEK293 cells	RS09		MERS	[79]
AnyGo Technology (Beijing, China)	S1-Fc		CHO cells	AD20Gold^+^ or CFA and AD11.10		RBD-Fc	[143]
IMVA-HB/IDMIT, VRI, Inserm (Fontenay-aux- Roses/Creteil/Paris, France)	A vaccine that targets the SARS-CoV-2 RBD to the CD40 receptor (αCD40.RBD)		CHO cells	Poly-IC		αCD40.HIVenv vaccine based on HIV envelope gp140 glycoprotein	[144]
LinkinVax, VRI, Inserm (Paris/Creteil, France)	A vaccine that targets antigens based on conserved T- and B-cell epitopes from the S and N proteins to the CD40 receptor (CD40.CoV2)		CHO-S cells	Poly-IC		αCD40.RBD vaccine	[144,145]
Sun Yat-Sen University, Institute of Human Virology (Guangzhou, China)	Ferritin nanoparticles conjugated to the SARS-CoV-2 RBD and HR (SpyTag/SpyCatcher system)		CHO-S cells	SAS		*H. pylori* ferritin-based influenza nanoparticle vaccines (NCT03186781 and NCT03814720)	[146]
Lomonosov Moscow State University (Moscow, Russia)	Recombinant antigens based on the RBD and the highly conserved antigenic fragments of the S2 subunit		*E. coli*	TMV SPs		Rubella and Anthrax	[43,147,148,149]

## Data Availability

Not applicable.
